# Y-type hexagonal ferrite-based band-pass filter with dual magnetic and electric field tunability

**DOI:** 10.1038/s41598-023-28279-8

**Published:** 2023-01-20

**Authors:** Maksym Popov, Yuzan Xiong, Igor Zavislyak, Hryhorii Chumak, Oleksandr Fedorchuk, Sujoy Saha, Rao Bidthanapally, Hongwei Qu, Michael R. Page, Gopalan Srinivasan

**Affiliations:** 1 Institute of High Technologies, Taras Shevchenko National University of Kyiv, Kyiv, Ukraine; 2 Electrical and Computer Engineering Department, Oakland University, Rochester, MI 48309 USA; 3 Solid State Chemistry Department, V.I. Vernadskii Institute of General and Inorganic Chemistry, Kyiv, Ukraine; 4 Physics Department, Oakland University, Rochester, MI 48309 USA; 5 Materials and Manufacturing Directorate, Air Force Research Laboratory, Wright-Patterson Air Force Base, Dayton, OH 45433 USA

**Keywords:** Materials science, Physics

## Abstract

This work is on the design, fabrication and characterization of a hexagonal ferrite band-pass filter that can be tuned either with a magnetic field or an electric field. The filter operation is based on a straight-edge Y-type hexagonal ferrite resonator symmetrically coupled to the input and output microstrip transmission lines. The Zn_2_Yfilter demonstrated magnetic field tunability in the 8–12 GHz frequency range by applying an in-plane bias magnetic field *H*_*0*_ provided by a built-in permanent magnet. The insertion loss and 3 dB bandwidth within this band were 8.6 ± 0.4 dB and 350 ± 40 MHz, respectively. The electric field *E* tunability of the pass-band of the device was facilitated by the nonlinear magnetoelectric effect (NLME) in the ferrite. The *E*-tuning of the center frequency of the filter by (1150 ± 90) MHz was obtained for an input DC electric power of 200 mW. With efforts directed at a significant reduction in the insertion loss, the compact and power efficient magnetic and electric field tunable Zn_2_Y band-pass filter has the potential for use in novel reconfigurable RF/microwave devices and communication systems.

## Introduction

Frequency selective components such as resonators, band-pass or band-stop filters are crucial parts of RF and microwave systems, including communication systems, radars, measurement equipment etc.,^1–3^. These components are used to either select or suppress a specific portion of the frequency spectrum of the passing signal and modify it according to the desired pattern. Specifically, band-pass filters are used for selective transmission of signals with reasonably low loss and simultaneously suppress the signals outside this band. The requirements for the filter bandwidth depend on specific application: a pre-selector in a spectrum analyzer may require a rather narrow span whereas an ultra-wideband communication system may need components with a large bandwidth. A particular avenue to improve the performance and add new capabilities to the abovementioned systems is making them reconfigurable (frequency agile) which will require the development of tunable subcomponents^3,4^.

Band-pass filters with electronically controlled transfer functions were developed using dielectric resonators with semiconductors or ferroelectric constituents^5,6^, printed circuit boards with varactors, pin-diodes or MEMS^7–9^, cavity filters with adjustable gaps^10,11^ etc. Among other technologies, filters based on spin-wave excitations have shown a potential for realizing components that are compact, planar, and tunable^12–15^. They make use of low-loss ferrites and, consequently, their microwave properties are tunable in a wide frequency range with a source of variable magnetic field. In some configurations these filters also demonstrate strong nonreciprocal characteristics, thus combing the properties of a filter and an isolator^15,16^. Those filters were based on either magnetostatic wave (MSW) propagation in tapered planar ferrite structures^12^ or MSW modes in ferrite samples with straight edges^16,18^. Despite such advantages, the need for a source of a tunable magnetic field presents some challenges in terms of device size, weight and power consumption. This has led to efforts to develop ferrite-based components with alternative ways of frequency tuning. Recent efforts in this regard include electric field tuning of resonators and filters utilizing strain mediated coupling in ferrite-piezoelectric composites^17–19^ as well as variation of dielectric constant in ferrite-ferroelectric composites^20,21^.

Both low-loss spinel and hexagonal ferrites are ideal for use in high frequency devices^22–25^. Early works on tunable ferrite filters mainly dealt with the use of ferrite spheres^24,25^. They demonstrated desired characteristics such as low insertion loss, yet the typical design of such filters made them incompatible for integration with the planar semiconductor device technologies. Then, by virtue of developments in thin-film growth technology, a combination of stripline transmission line with ferrite films became possible which resulted in devices that were compatible with the hybrid and monolithic microwave integrated circuits^26–28^.

This work is on a dual magnetic field and electric field tunable Y-type hexagonal ferrite filter. The magnetic field *H* tuning of the filter was accomplished with a built-in permanent magnet and the electric field *E* tuning was facilitated by the recently reported nonlinear magnetoelectric (NLME) effect in M- and Y-type hexagonal ferrites^22,23^. Application of a pulsed or DC electric field to the ferrite was found to result in variations in the magnetization and magnetocrystalline anisotropy field and the changes in these order parameters were proportional to *E*^*2*^. Although a first principles theory for the non-linear ME effects is lacking at present, the likely cause of the modification of magnetic parameters in an electric field and the consequent tuning of FMR is related to hopping type electrical conduction current in the ferrites. Since the Fe ions involved in the conduction process are effectively excluded from contributing to magnetic interactions, one may anticipate weakening of both the super-exchange interaction and the strength of spin–orbit coupling. This leads to variations in the magnetization and anisotropy field and results in tuning of the frequency of magnetic modes^22,23^. The changes in the magnetic parameters resulted in tuning of the ferromagnetic resonance (FMR) frequency, with the frequency changes being proportional to the DC electric power applied to the sample. Thus, the use of the hexagonal ferrite as the coupling element in a band-pass filter resulted in *E* or current tunability of the device. In the following sections we discuss the filter design and its *H*- and *E*-tuning characteristics.

## Methods

**Figure 1:**
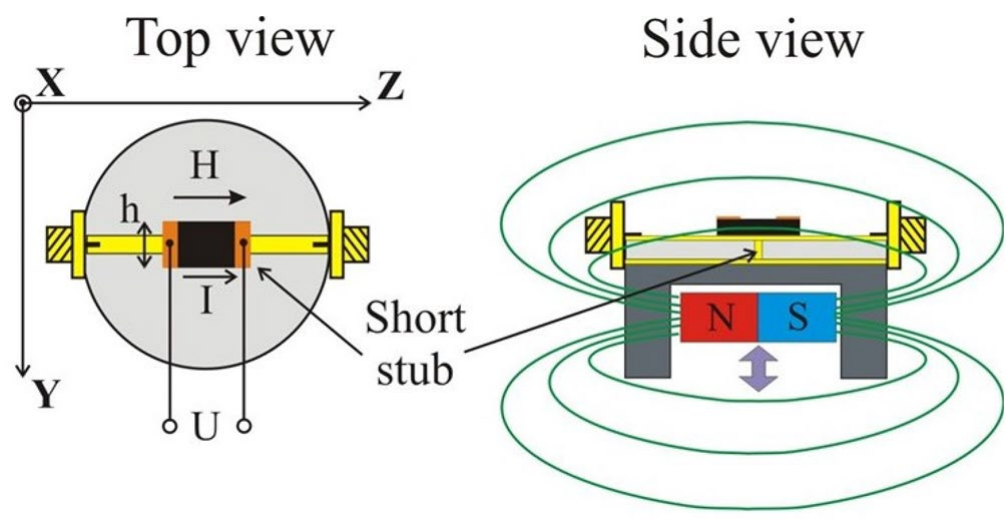
Schematics of the ferrite-based band-pass filter prototype showing the position of Zn_2_Y resonator on top of the short-circuited microstrip line and a permanent magnet located inside the filter frame to apply a bias magnetic field.

The schematics of the filter are shown in Fig. 1. It consists of a microstrip line with 50 Ohm impedance manufactured using 0.01-inch-thick RT/Duroid ® 5880.In the middle of the stripline a short circuit was created with a vertical copper stub that was soldered to both signal line and bottom ground. The width of the stub was the same as the width of the microstrip line.

Such configuration provides the necessary isolation between the input and output ports by at least 20 dB in the frequency range upto13 GHz. A ferrite straight-edge resonator made from single-crystalline Y-type hexaferrite,Ba_2_Zn_2_Fe_12_O_22_^29^ (labeled as Zn_2_Y),with lateral dimensions of 1.15 × 2.15 mm^2^ and thickness *S* = 150 μm was used in the device. The specimen was cut in such way that the *c*- axis was normal to the sample plane. It was positioned on top of the stripline and oriented with longer side along the microstrip. Two platinum electrodes were deposited on opposite edges of the sample, allowing the application of a voltage to establish an electric field parallel to the sample plane. Thus, the resulting flow of DC electric current through the ferrite is in the basal plane. Our recent study on NLME in Zn_2_Y indicated the strongest interaction for in-plane current^30^. A thin 15 μm sheet of isolating material (mica) was placed between the sample and the microstrip line to avoid electrical contact.

A disk-shaped permanent magnet as shown in Fig. 1 was used to generate the necessary bias field magnetic field *H*_0_ parallel to the sample plane as indicated by the magnetic force lines. The field *H*_*0*_ was found to be uniform over the entire volume of the ferrite and the field strength could be varied by adjusting the separation between the magnet and the device ground plane. When the ferrite resonator is magnetized to saturation, it is able to support multiple magnetostatic wave (MSW) modes^31,32^, with the resonance frequencies depending on *H*_*0*_, saturation magnetization, and the anisotropy field values, as discussed below. The MSW mode excited in turn will couple the input microwave power to the output microstrip line. The largest signal-to-resonator coupling is expected for the lowest-order MSW mode, which has the most uniform spatial distribution of the high-frequency magnetization. On the contrary, the signals with frequency much different from resonance will not affect the resonator and should be just reflected from the short circuit at the middle of the line, forming the rejection band of the filter. From the above considerations, the filter bandwidth is anticipated to be proportional to the resonator linewidth and hence inversely proportional to loaded Q-factor of the resonator mode. In such configuration the transverse orientation of microstrip line microwave field *h* facilitated effective excitation of magnetic oscillations in ferrite resonator. It was experimentally confirmed that the lowest insertion losses for the device were obtained for the magnetic field just along the signal line.

### Theoretical analysis

#### Magnetostatic waves is ferrite films

Since the ferrite slab is tangentially magnetized, it can support two types of magnetostatic waves, namely magnetostatic surface waves (MSSW) and backward volume waves (MSBVW)^31,32^. The former is known to propagate in the direction perpendicular to bias magnetic field whereas for the latter propagation direction is parallel to the bias magnetic field. Based on our filter design it is reasonable to assume that the in-band transmission for the filter are facilitated by the MSBVW propagating along the sample, just in the overall direction of microwave energy flow.

The dispersion relation for MSBVWs for a ferrite film with thickness *S* and a saturating bias magnetic field in the *z* direction is given by^32^ (see Fig. 1 for the axes directions)

1 $$ \tan \left( {\frac{{k_{z} S}}{{2\sqrt { - \mu } }}} \right) = \sqrt { - \mu } $$

where *μ* < 0is the diagonal term of the microwave magnetic permeability tensor for the ferrite. Due to the periodic nature of tangent function Eq. (1) has multiple solutions which may be labeled by increasing integer numbers^32^.

2 $$ \begin{gathered} k_{z} S = 2\sqrt { - \mu } (m\pi + \arctan \sqrt { - \mu } ),\quad m = 0,{1},{2} \ldots \hfill \\ k_{z} S = 2\sqrt { - \mu } \left( {n\pi - \arctan \frac{1}{{\sqrt { - \mu } }}} \right),\quad n = {1},{2} \ldots \hfill \\ \end{gathered} $$

Among those modes, the lowest-order one, with *m* = 0, has the simplest (most uniform) high-frequency magnetization distribution along the sample thickness and is easily excited by (nearly) uniform RF magnetic field. The presence of metal screens near the ferrite film will affect the MSW propagation characteristics. However, this effect is most pronounced in the case of MSSW^33^, whereas the BVW modes are less affected. The upper and lower boundaries of the BVW frequency band remain the same, and only the dispersion curve slope $\partial f/\partial k_{z}$ will vary^32^. Moreover, a single side metallization just results in effective increase of film thickness from *S* to 2*S* leaving rest of Eq. (2) unchanged.

For the band-pass filter the transmission properties are determined by the MSW mode(s) excited in the central, unmetallized part of the resonator. Since the resonator is directly on top of the conducting microstrip line, the best theoretical approximation is the model for single-side metalized ferrite film. The dispersion equation is then

3 $$ k_{z} S = \sqrt { - \mu } (\arctan \sqrt { - \mu } ) $$

and the mode frequencies are obtained by substituting $k_{z} = k_{z,p} = p\pi /L$, where *p* = 1,2,3… and *L* is the effective length of the ferrite resonator in the direction of signal propagation. Since *L* >  > *S*, the term *k*_*z*_*S* for lowest-order modes should be rather small, and so |*μ*| also will be small. Expanding the right-hand side of Eq. (3) in the limit |*μ*|< < 1 one obtains

$$ k_{z,p} S \approx - \mu $$

Finally, after substituting the expression for *μ* for the ferrite with easy-plane anisotropy^32^, we obtain in explicit form

4 $$ f_{p} \approx \gamma \sqrt {H_{0} (H_{0} + H_{a} + 4\pi M_{S} ) - (4\pi M_{S} H_{0} )k_{z,p} S} $$

Here *γ* is the gyromagnetic ratio, *H*_0_ is the bias magnetic field, 4*πM*_*S*_ is the saturation magnetization of the ferrite, and *H*_a_ is the magnetocrystalline anisotropy field. For very small values of the normalized wavevector $k_{z,p} S \to 0$ and resonator eigenfrequency tends to the known expression for the uniform FMR mode in easy-plane ferromagnet, namely^34^

5 $$ f_{0} = \gamma \sqrt {H_{0} (H_{0} + H_{a} + 4\pi M_{S} )} . $$

As it was demonstrated in Ref.^23^ the application of DC electric current to hexaferrite sample results in strong modification of “effective” magnetization $4\pi M_{eff} = H_{a} + 4\pi M_{S}$ which is almost linearly proportional to the applied electric power *P*:

6 $$ \Delta (4\pi M_{eff} )(P) = C\frac{\rho }{V}P $$

where *ρ* is the sample’s specific resistivity, *V* is its volume and *C* is the term that includes third- and forth-order ME tensor components as well as unmodified values of *H*_*a*_ and 4π*M*_*S*_. From Eq. (4) it follows that such ME modification of magnetic parameters will lead to changes in the resonator mode frequency and thus to the tuning of the pass-band of the filter.

#### Simulation results

**Figure 2:**
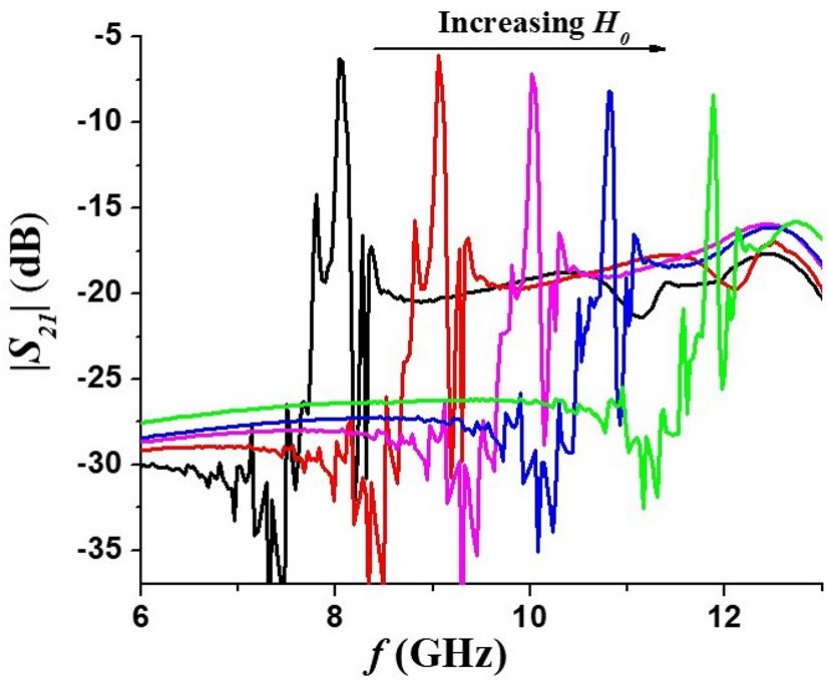
Simulation of the proposed band-pass filter transmission properties in the X-band.

The designed band-pass filter characteristics were simulated with Ansoft High Frequency Structure Simulator (HFSS) software and the results are presented in Fig. 2. The magnetic and electric parameters used for simulation were the following: 4*πM*_*S*_ = 2300 G^23^, dielectric constant ε = 12, and ferromagnetic linewidth Δ*H* = 15 Oe^29^. Since HFSS cannot correctly account for the easy plane uniaxial anisotropy, the value of *H*_*a*_ was not included in the simulation. Instead, a model of isotropic ferrite was used and magnitude of external magnetic field was selected in such way to provide the required filter center frequency. Therefore, values of *H*_0_ are not given in Fig. 2.

From the results in Fig. 2 we can expect a minimum insertion loss of 8–11 dB with the 3-dB bandwidth of ≈ 75–85 MHz. Also note the presence of multiple spurious modes with frequencies below the main resonance. They are consistent with Eq. (4) which predicts that higher order modes (those with larger *k*_z,p_) will possess a lower frequency. For the reason stated above, these results cannot be treated as a complete simulation; however, as will be shown below, many general trends of the filter parameters are reproduced correctly.

## Results and discussion

**Figure 3:**
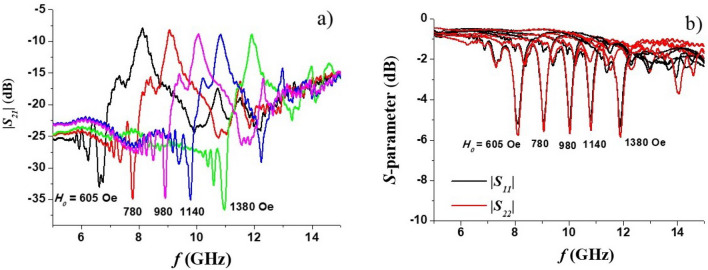
The measured transmission characteristics (**a**) and reflection coefficients (**b**) of the band-pass filter under different bias fields (the *H*_*0*_ values were inferred from Fig. 2 in Ref^23^.)

The *S*-parameters of the filter were measured with a vector network analyzer (Agilent PNA N5230A). The magnitudes of the scattering matrix parameters |*S*_21_| and |*S*_12_|, as well as the reflection coefficient*S*_*11*_, were measured under a series of bias magnetic fields. Representative data are shown in Fig. 3. During these measurements no DC voltage was applied to the sample. Accurate values of the bias field *H*_0_ could not recorded due to the following reasons. The magnetic field was produced by a permanent magnet. When the bias field is measured outside.

**Figure 4:**
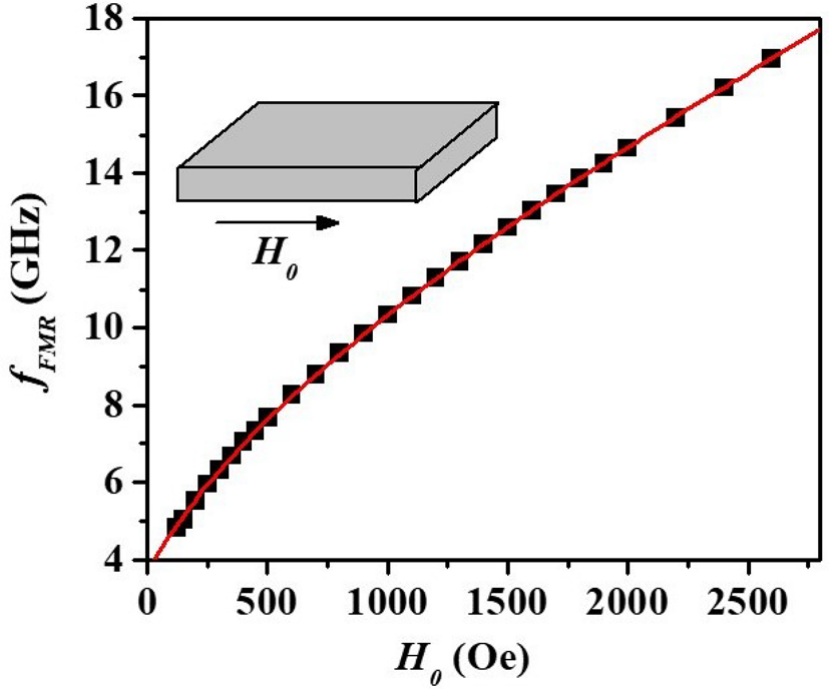
Resonance mode frequency as a function of bias field *H* for Zn_2_Y. These data were used for estimation of *H*_*0*_ values in Fig. 3

The sample, the difference between the value registered by the Gaussmeter and the actual field at the position of ferrite resonator would be different for any meaningful comparison. We therefore estimated *H*_*0*_ values by using data in Fig. 4 on resonance frequency as a function of the bias field produced by an electromagnet for a sample of Zn_2_Y of identical dimensions as the platelet used in the filter^23^. The bias fields *H*_0_ in Fig. 3 for the profiles in Fig. 3 were estimated from these data. The central frequency of the pass band in Fig. 3 ranged from 8 to 12 GHz (X-band) for the bias field interval of ≈ 550–1400 Oe.

Comparing profiles in Fig. 3 with the results of simulation in Fig. 2 one notices that the general shape of the measured transmission characteristics complies with the simulation results. This includes the presence of multiple spurious resonances at frequencies below the pass band and notable deep notches on both sides of the pass-band. The frequencies of spurious modes and notches in Fig. 2 are not consistent with profiles of Fig. 3 which is due to somewhat deficient nature of the simulation model as explained earlier. The values of the insertion loss over the pass-band in Fig. 3 and simulation results are in a good agreement.

The main difference between the simulation and experiment is in the transmission bandwidth. The measured bandwidths in Fig. 3 are approximately5 times larger than the simulation results. We attribute this to nonuniform dc bias field inside the ferrite resonator. In simulation, the internal magnetic field is assumed to be homogeneous whereas in the experiment the bias field from permanent magnet will be slightly nonuniform both along the thickness of the sample and in its plane. Moreover, it is known that in resonators of non-ellipsoidal shape the *internal* magnetic field would be nonuniform even if they are placed in perfectly uniform *external* field^35^. The nonuniform bias field in Zn_2_Y film broadens the width of the pass-band, since the magnetic resonance will occur at different frequencies at different positions of the sample. Also, the actual ferromagnetic resonance linewidth in the sample may be somewhat larger than assumed Δ*H* values for the simulation.

**Figure 5:**
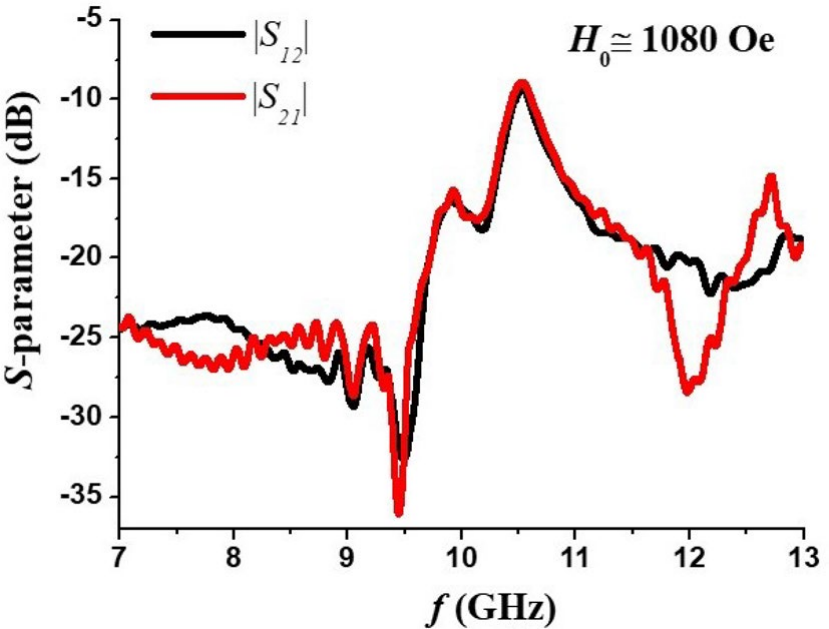
Profiles of transmission characteristics showing nonreciprocity for the filter. *H*_*0*_ value was extracted from data in Fig. 4.

Figure 5 shows profiles of |*S*_21_| and |*S*_12_| versus frequency and the band-pass characteristics of the filter shows slight nonreciprocity that could be attributed to a nonsymmetrical position of the resonator with respect to the short-circuit metal slab, and thus to non-equal coupling of the resonator modes with the electromagnetic signals coming from the two opposite directions.

**Figure 6:**
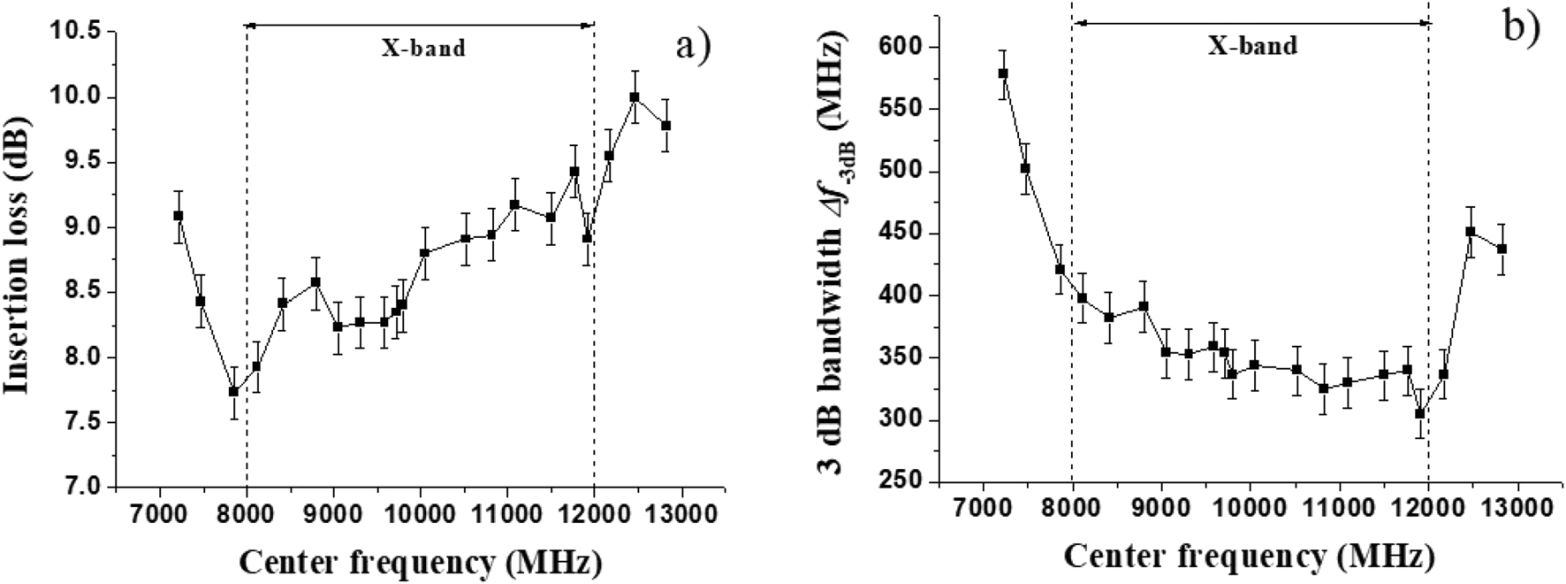
(**a**) Insertion loss and (**b**) 3-dB bandwidth for the band-pass filter in the X-band.

The insertion loss and 3-dB bandwidth of the filter measured with the change of the center frequency of the filter (for bias magnetic field of ≈ 600–1600 Oe) are plotted in the Fig. 6.

One notices that within the X-band the insertion losses are in the 8–9 dB range. From the reflection coefficients shown in the Fig. 3 one may attribute such losses, in part, to the poor matching between the resonator and both input and output transmission lines. A capacitive matching circuit on both sides of the ferrite sample might reduce the insertion loss. The filter bandwidth is rather large in comparison, for example, with yttrium-iron garnet (YIG) filters. The contributing factor, besides possible bias field nonuniformity, is the large magnetic losses in Zn_2_Y. It follows from Ref.^36^ that the full 3 dB linewidth of the two-side coupled resonator is given by

$$ \Delta f_{ - 3dB} = f_{c} \frac{{(1 + K_{1} + K_{2} )}}{{Q_{0} }} $$

where *f*_*c*_ is the center frequency, *K*_*i*_ > 0 are the coupling coefficients with the input and output transmission lines, and *Q*_0_ is the unloaded quality factor that accounts for internal losses in resonator. In turn, *Q*_0_ is determined by the internal magnetic field and the FMR linewidth. For in-plane magnetized film $Q_{0} = \frac{{H_{0} }}{\Delta H}$. Therefore, for the comparable values of *f*_*c*_ and *K*_*i*_ resonator made with smaller Δ*H* material will have a proportionally narrower linewidth. Since the lowest Δ*H* values for single-crystal Zn_2_Y are on the order of 10–15 Oe^29^ whereas the best YIG films have Δ*H* ~ 1 Oe, it is not surprising that the narrowest 3 dB linewidths of 10-20 MHz for YIG–based filters^12,13,38^ will be unattainable for Zn_2_Y filters.

**Figure 7:**
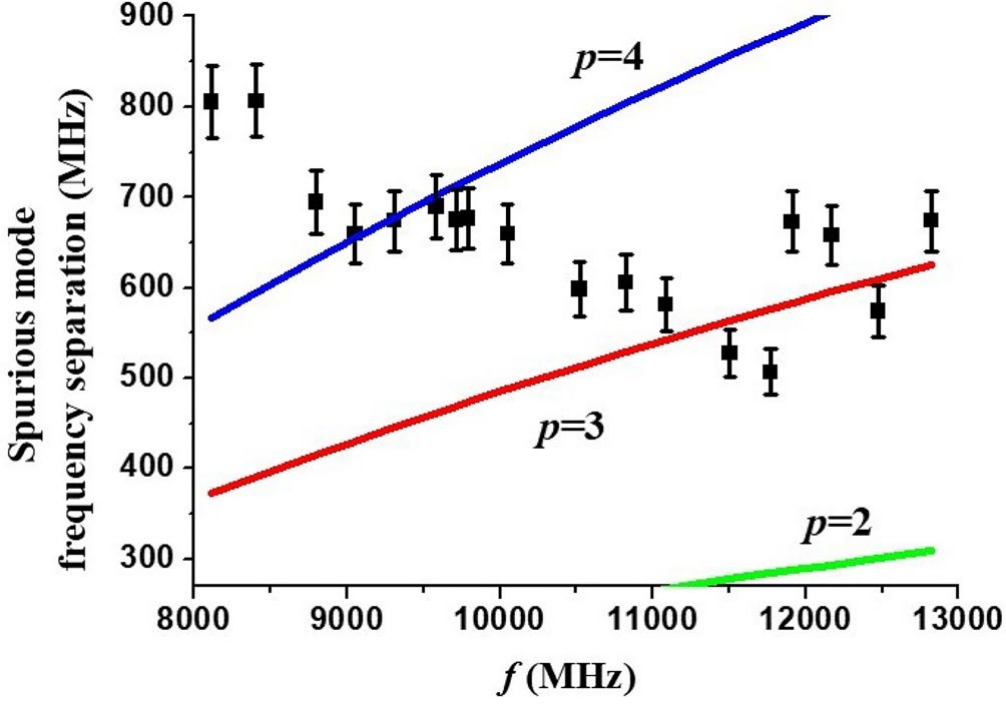
The frequency separation between band-pass filter center frequency and the largest spurious mode. Dots—experimental data, solid lines—theory.

From Fig. 3 it can be seen that the filter characteristics show an out-of-band spurious resonance located below the main pass-band. It was attributed to one of the higher-order BVW modes that are excited in the ferrite resonator. Indeed, backward volume waves are known for having a negative slope of dispersion curve i.e. the mode frequency decreases with increasing wavenumber^32^.Thus, the higher order modes with larger *k*_z,p_ will have a progressively lower eigenfrequencies. The same conclusion can be inferred explicitly from Eq. (4). Figure 7 shows data on the frequencies of spurious peaks with the largest magnitude extracted from the recorded filter characteristics. For the sake of convenience, only the frequency difference between pass-band center frequency and peak frequency is given. Theoretical curves for the frequency separation between main (*p* = 1) and higher-order (*p* = 2, 3…) modes, namely, |*f*_1_ – *f*_2_|, |*f*_1_ – *f*_3_|, |*f*_1_ – *f*_4_|, calculated using Eq. (4) and Zn_2_Y magnetic parameters are also shown Fig. 7. For the calculations an effective resonator length value *L* = 1.7 mm was used. It is smaller than the physical length of the sample and accounts for the presence of metallization pads on both edges of ferrite which reduce the resonator’s actual dimensions. It can be seen that in lower half of the X-band the largest spurious peak may be identified as *p* = 4 higher-order mode, whereas in the upper half it is most likely the *p* = 3 mode.

**Figure 8:**
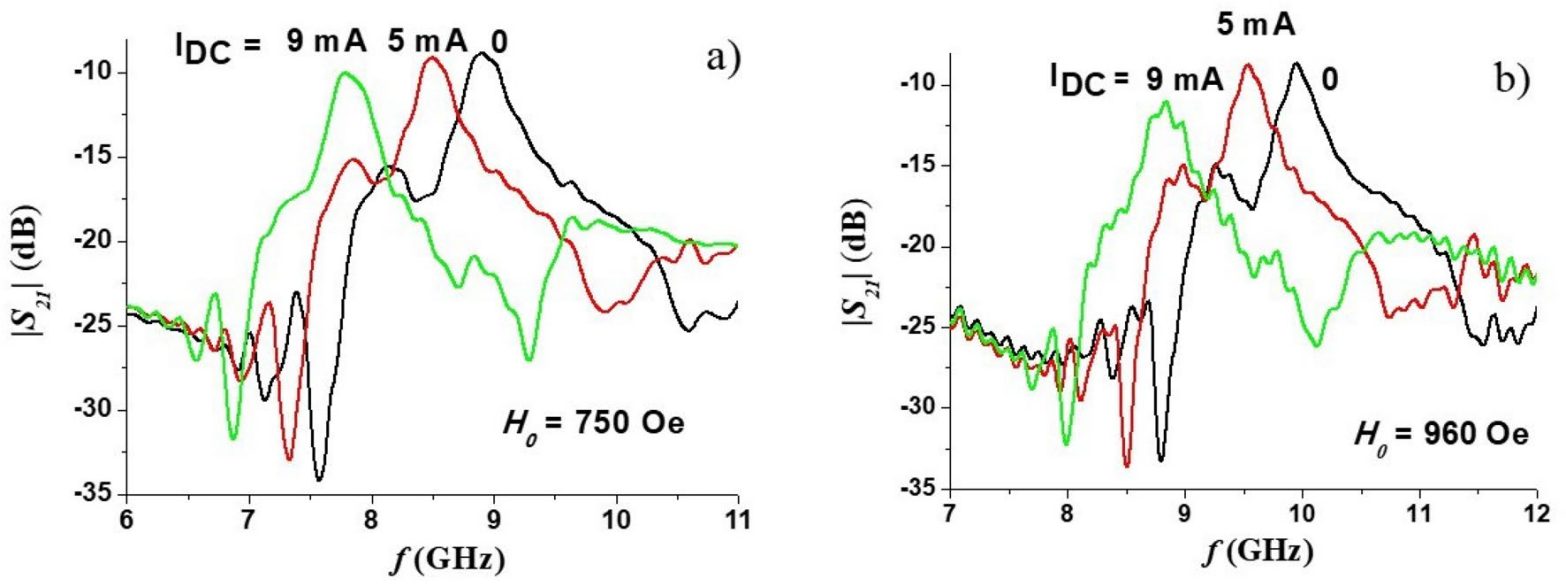
Pass-band electrical tuning characteristics of the band pass filter by applied DC current for (**a**) *H*_*0*_ = 750 Oe and (**b**) *H*_*0*_ = 960.

The dual tunability of the band-pass filter was demonstrated by applying a DC current along the sample basal plane and recording the non-linear ME effect induced changes in the transmission characteristics. The small-signal resistance of the sample was *R* = 6.7 kΩ at room temperature. Due to the semiconductor nature of the ferrite the linear dependence between applied voltage *U* and current *I* maintained only in a limited range of *I* and voltage-current characteristic reached saturation for *I* > 10 mA. This sets the limit for the maximum applied current. Representative profiles for tuning the pass-band by applied DC currents are presented in Fig. 8. The profiles show a downshift in the center frequency due to the current. This is expected based on results of our studies in hexagonal ferrites that showed a decrease in the effective magnetization $4\pi M_{eff}$ leading to a decrease in the mode frequency^23^.

**Figure 9:**
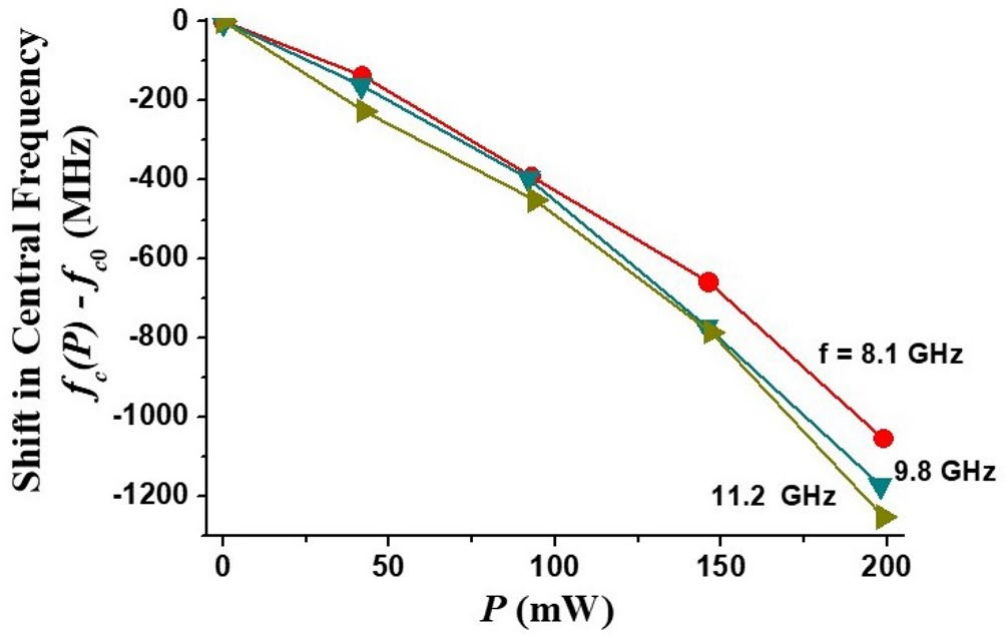
Shift in the pass filter center frequency as a function of the applied electric power.

The dependence of the center frequency of the filter on the applied input power *P* = *UI* for different bias magnetic fields are shown in Fig. 9. A current-tunable center frequency shift of more than 1 GHz is routinely obtained for *P *≈ 200 mW. Although the *4πM*_*eff*_ dependence on *P* is expected to be linear, the frequency shift dependence on *P* is not linear due to the nonlinear relation between *f*_*p*_ and $4\pi M_{eff}$ in Eq. (4). Also, note that the absolute frequency shift is larger for the frequencies around 12 GHz in comparisons with frequencies close to 8 GHz. It is again in accordance with Eq. (4) since larger frequencies mean larger applied field *H*_0_ which, being a prefactor for $4\pi M_{eff}$, enhances its effect.

**Figure 10:**
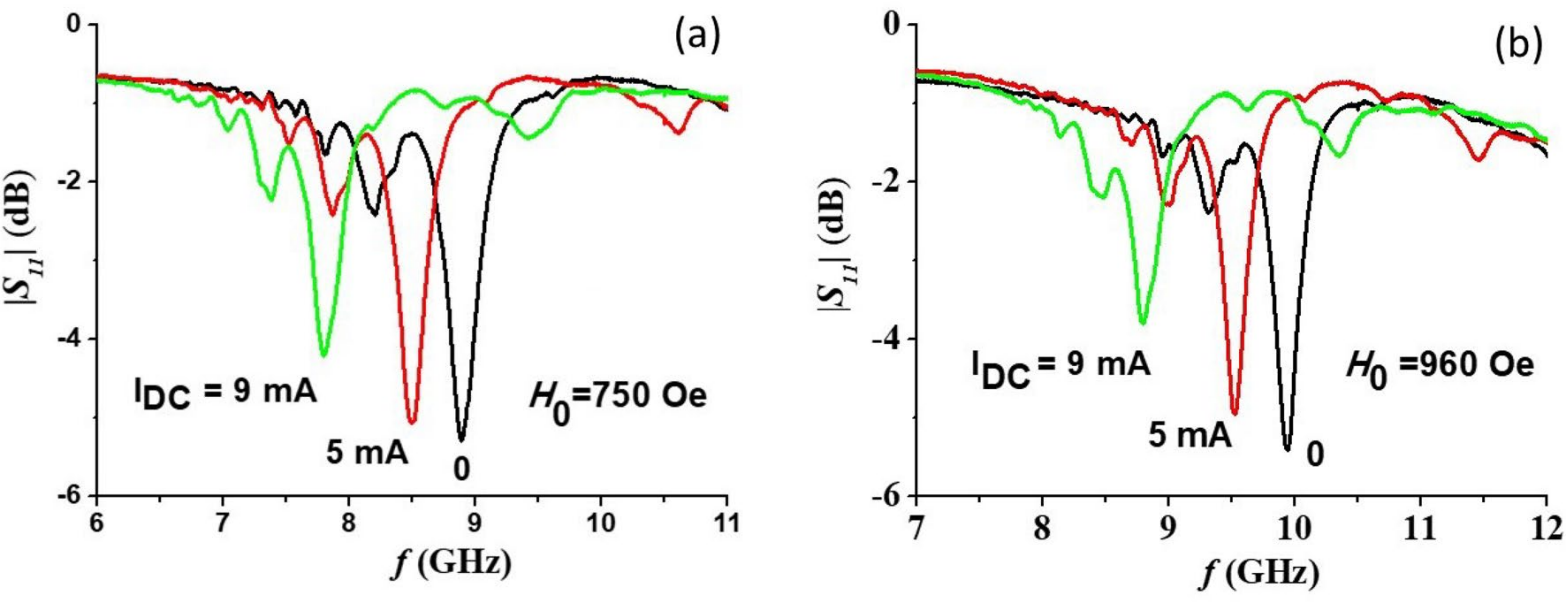
Return loss *S*_*11*_ as a function of frequency *f* for a series of DC current for (**a**) *H*_*0*_ = 750 Oe and (**b**) *H*_*0*_ = 960 Oe.

We carried out measurements of *S*_*11*_ vs *f* for the filter and the results are shown in Fig. 10. The electrical tuning range of the center frequency of absorption peak is almost the same as was measured from the transmission data in Fig. 8. The width of the reflection peak also demonstrates a similar tendency as for *S*_*21*_, namely, for relatively small currents the linewidth shows very little changes, whereas for large currents broadening of peaks are observed in Fig. 10. Therefore, upon electrical tuning variations in both the center frequency and linewidth show similar characteristics in *S*_*11*_ and *S*_*22*_ versus frequency data.

Finally, it is seen that for relatively small currents the filter mostly retains the insertion loss and bandwidth values, whereas for large current both parameters deteriorate. This may be due to the changes of complex microwave impedance of ferrite material which are known to take place in semiconductors with the increase of applied constant voltage^39^. The impedance changes will affect matching conditions and increased internal losses will decrease the unloaded Q-factor.

Tuning speed is a key parameter for the filter. Magnetic tuning of the filter was slow since it involved manual repositioning of the permanent magnet. We reported recently on electrical tuning of a Zn_2_Y device (Fig. 7 in Ref^23^.). Data were obtained on shift in the FMR frequency as a function of time in steps of 0.1–0.2 s for a series of currents through the device. These data showed frequency small frequency shifts of 200–300 MHz within a few tenths of a second and the device attained saturation values of the shifts within 1–2 s. For a given frequency, the tuning speed progressively increased with increasing magnitude of tuning currents. In general, such tuning speeds may be explained by the time required to charge the capacitor formed by contact electrodes on the ferrite sample.”

Since the Joule heating of the ferrite sample is expected to result in a decrease in the magnetization and a change in the FMR frequency, it is important to compare any shift due to heating with NLME effects. In our recent work on NLME in Zn_2_Y, we reported on the influence of sample heating on the shift in the magnetic mode frequencies due to Joule heating^23^. A sample of similar dimensions as the resonator used in this was used. A continuous DC current (power *P* = 109 mW) was applied to the sample and the sample temperature and frequency shift were measured for a duration of 600 s. As soon as the DC current was applied the FMR frequency decreased shifted by ~ 900 MHz that was attributed to NLME (Fig. 8 in Ref^23^). The sample attained thermal equilibrium in 10 min and its temperature increased by 1 C (Fig. 9 in Ref^23^) and the overall shift in FMR frequency was ~ 925 MHz. Thus, the shift that could be attributed to Joule heating was 25 MHz and was negligibly small compared NLME induced shift. For the sample used in this study one expects a change in sample temperature by ~ 2 °C for input DC power of ~ 200 mW and a subsequent shift in FMR frequency by ~ 50 MHz due to heating versus a shift of ~ 1 GHz due to NLME.

## Conclusions

In summary, a prototype of dual-tunable band-pass filter based on a Y-type hexagonal ferrite straight-edge resonator is presented, fabricated and tested. The filter operation is based on the excitation of the BVMSW modes which is then coupled to the input and output microstrip transmission lines. The dual tunability comes from the fact that resonance mode frequency can be adjusted by either varying the value of external bias magnetic field or supplying a DC current in ferrite basal plane. Both techniques were demonstrated experimentally.

The measured filter specifications are the following: the pass band center frequency is tunable within the X-band (8–12 GHz) with a bias magnetic field of approx. 500–1500 Oe applied in the filter plane along the signal line; the insertion loss and 3 dB bandwidth within this band are 8.6 ± 0.4 dB and 350 ± 40 MHz, respectively. A means to decrease the linewidth (by providing spatially uniform bias field) and improve the insertion losses (by adding a matching network for optimal coupling) were outlined.

The maximum electric tunability of the filter in the X-band amounted to—(1150 ± 90) MHz which is 10–12% of the center frequency. This tuning is achieved with applied electric power of 200 mW. The permanent magnet for the bias field in this design is integrated with the filter frame, thus, eliminating the need for an external electromagnet. It makes possible a current-only control of the passband center frequency in the stated above frequency range after a reference center frequency is set by the permanent magnet arrangement. Such compact and low power consumption tunable band-pass filter design can find applications in various frequency agile RF and microwave systems.

## Data Availability

The datasets used and/or analysed during the current study available from the corresponding author on reasonable request.
